# Neanderthal-Derived Genetic Variation Shapes Modern Human Cranium and Brain

**DOI:** 10.1038/s41598-017-06587-0

**Published:** 2017-07-24

**Authors:** Michael D. Gregory, J. Shane Kippenhan, Daniel P. Eisenberg, Philip D. Kohn, Dwight Dickinson, Venkata S. Mattay, Qiang Chen, Daniel R. Weinberger, Ziad S. Saad, Karen F. Berman

**Affiliations:** 1 Section on Integrative Neuroimaging, Clinical and Translational Neuroscience Branch, National Institute of Mental Health Intramural Research Program, National Institutes of Health, Bethesda, MD USA; 2 Psychosis and Cognitive Studies Section, Clinical and Translational Neuroscience Branch, National Institute of Mental Health Intramural Research Program, National Institutes of Health, Bethesda, MD USA; 3 Lieber Institute for Brain Development, Johns Hopkins Medical Campus, Baltimore, MD USA; 4 Department of Neurology, Johns Hopkins University School of Medicine, Baltimore, MD USA; 5 Departments of Psychiatry, Neuroscience, and the McKusick-Nathans Institute of Genetic Medicine, Johns Hopkins School of Medicine, Baltimore, MD USA; 6 Scientific and Statistical Computing Core, National Institute of Mental Health Intramural Research Program, National Institutes of Health, Bethesda, MD USA

## Abstract

Before their disappearance from the fossil record approximately 40,000 years ago, Neanderthals, the ancient hominin lineage most closely related to modern humans, interbred with ancestors of present-day humans. The legacy of this gene flow persists through Neanderthal-derived variants that survive in modern human DNA; however, the neural implications of this inheritance are uncertain. Here, using MRI in a large cohort of healthy individuals of European-descent, we show that the amount of Neanderthal-originating polymorphism carried in living humans is related to cranial and brain morphology. First, as a validation of our approach, we demonstrate that a greater load of Neanderthal-derived genetic variants (higher “NeanderScore”) is associated with skull shapes resembling those of known Neanderthal cranial remains, particularly in occipital and parietal bones. Next, we demonstrate convergent NeanderScore-related findings in the brain (measured by gray- and white-matter volume, sulcal depth, and gyrification index) that localize to the visual cortex and intraparietal sulcus. This work provides insights into ancestral human neurobiology and suggests that Neanderthal-derived genetic variation is neurologically functional in the contemporary population.

## Introduction

The most recent evolutionary relative of *H. sapiens*, *H. neanderthalensis*, represents a lineage associated with archeological samples (e.g., tools, pigments) suggesting substantial cognitive achievements^1^, and is typically contrasted with anatomically modern humans by particular cranial features known from fossil remains^2^. The initial sequencing of the Neanderthal genome summarily unearthed an unprecedented ingress to Neanderthal biology, resulting not only in a catalogue of shared ancestral alleles and divergent variation relative to *H. sapiens* but also evidence for gene flow from Neanderthals to modern humans^3, 4^, which has since been well-supported^5–8^. This introduction of Neanderthal alleles most likely occurred in Eurasia, as modern humans ventured out of Africa, between 47,000 and 65,000 years ago^7^ and accounts for about 2% of the entire non-African human genome^8^. The functional implications of this observation, however, are not fully understood.

If these alleles are biologically meaningful for their modern bearers^5^, and by extension, for their extinct originators, individuals endowed with greater proportions of Neanderthal-derived sequence differences might be expected to harbor Neanderthal-like phenotypes. To test this hypothesis, rather than using an additive model in a genetic association analysis, we performed linear regressions of polygenic measures of Neanderthal-derived common variant data with MRI imaging data. Previous reports^3, 5^ have described methods for testing whether single nucleotide polymorhpisms (SNPs) are derived from admixture of modern humans with Neanderthals. Here, after computing an enhanced D statistic^9^, which measures the percentage of these alleles present in each individual (subsequently termed “NeanderScore”), we sought to identify whether variation was associated with regional changes in skull and brain shape. Because cranial morphology is a pivotal physical element distinguishing Neanderthals from other human lineages in fossil records, it provides an ideal phenotype to test our approach and the impact of our Neanderthal-derived genetic characteristics. We anticipated that some of the Neanderthal genetic variants responsible for skull development might be represented in our sample and, when sufficiently aggregated in higher NeanderScore individuals, that these variants would be associated with hallmark Neanderthal-like morphological biases, such as increased skull length^10, 11^ and posterolateral broadening of the cranium^2^.

As the distinctive cranial morphology of *H. neanderthalensis* is hypothesized to reflect underlying brain anatomy^12–16^, in addition to characterizing the relationship between NeanderScore and modern human cranial shape, we also sought to define contributions of Neanderthal-derived genetic variation to brain structure, as measured by gray- and white-matter volume, regional sulcal depth, and gyrification index. We further hypothesized that the morphometry of brain regions underlying the identified NeanderScore-associated cranial regions would also be associated with Neanderthal genetic load.

## Results

### Calculation of NeanderScore

We first examined common variant genotype data acquired from 223 healthy individuals of self-reported European-descent who also underwent magnetic resonance imaging (MRI) of the head. To ensure all participants were of similar genetic ancestry, multidimensional scaling of ancestry-informative genetic markers^17^ was performed on the entire sample. Two participants were found to be significantly different from the rest of the group and were excluded, while the remaining 221 individuals (average age 33.7 ± 10.6 years, range 18–60, 116 females) were retained for further analyses examining each individual’s percentage of Neanderthal-derived genetic material. Following previously reported methods^3, 5^, we identified 100,156 single nucleotide polymorphisms (SNPs) in our sample that are putatively derived from Neanderthals and then calculated an enhanced D statistic^9^, which determined the percentage that were present in each of the 221 individuals (NeanderScore). Consistent with previous reports^3, 5, 8^, an expectedly small but normally distributed percentage of Neanderthal sequence variation was present in our participants, with individuals showing an average NeanderScore of 5.4% (standard deviation = 0.38%, range = 3.9–6.5%, coefficient of variation = 0.070). It is important to note that the NeanderScore measure is a related but different measure than prior reports of Neanderthal admixture (as discussed below), and thus has a different mean and standard deviation than those in previous reports; however, our coefficient of variation is identical to those previously reported and indicates sufficient variability to pursue further quantitative trait analyses with the MRI data. Table 1 shows demographic information of the sample. As the high coverage Altai Neanderthal draft sequence was used as the Neanderthal genotypes for each SNP, we examined the subset of 715 SNPs overlapping with the exome data publically available and previously reported for the Vindija and El Sidron Neanderthal samples^18^, as well as for a Denisovan sample, to determine the degree these SNPs are specific to Neanderthals and maintained in other archaic samples (data acquired from http://cdna.eva.mpg.de/neandertal/exomes/). We found a high degree of concordance of the Altai sequence with the other Neanderthal samples (94.9% for Vindija and 93.8% for El Sidron). In contrast, the Denisovan sample showed a much lower concordance rate of 26.4%, suggesting both sensitivity and specificity of our measure to Neanderthal admixture. As more high coverage archaic samples become available, future work could further describe and investigate the implications of genetic variability in these samples. Additionally, to ensure that the NeanderScore variation observed was not related to possible subtle differences in the microarray chips used, we tested for differences in NeanderScore across these chips and found none (F(2,218) = 0.023, p = 0.98).

**Table 1:** Subject demographics for the entire group and subset of subjects used in the skull shape analysis, mean ± SD.

	N	Age	Sex	NeanderScore
**Entire Group**	221	33.7 ± 10.6 years range 18–60 years	105 Males/116 Females	5.39 ± 0.38% range 3.9–6.5%
**Skull Subgroup**	146	34.1 ± 10.4 years range 20–59 years	59 Males/87 Females	5.36 ± 0.39% range 3.9–6.5%

### Relationships between NeanderScore and Skull Shape

For the subset of our cohort with high-quality, full cranium acquisitions (N = 146, Table 1), we created a 3D skull model for each individual using a previously validated algorithm^19–21^, as well as a 3D template skull model created from the group average. After conducting a nine-parameter alignment between each individual’s model and the template model, we calculated the distance between each node of the template and the nearest node in each individual skull^22^. We then assessed correlations between these local shape values and NeanderScore.

**Figure 1:**
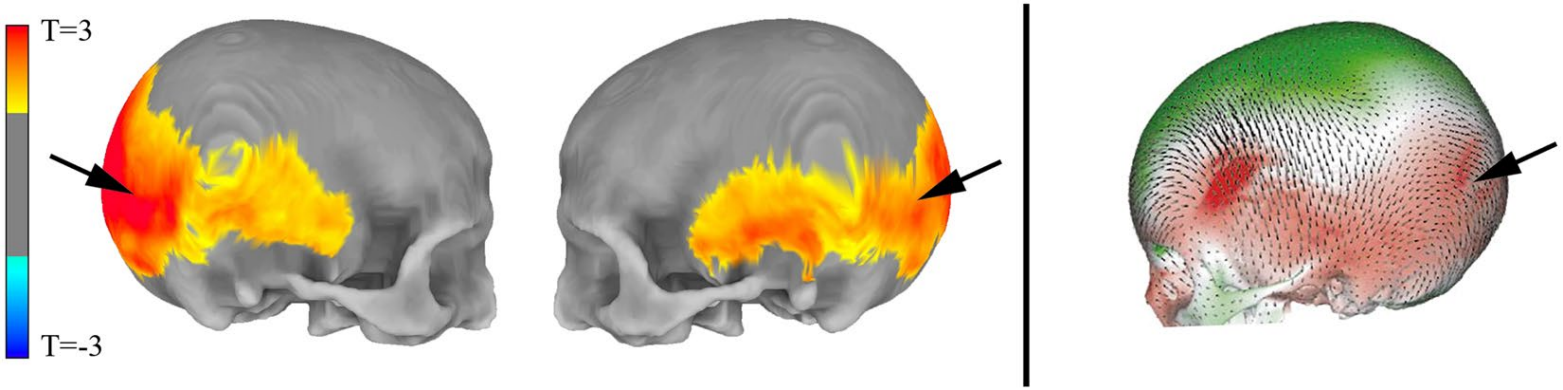
NeanderScore related skull shape changes. Associations between skull morphology and percentage of Neanderthal-derived SNPs (NeanderScore) in modern humans (Left), compared to differences between Neanderthal and modern human skulls (Right). Left panel shows regions with morphological changes, measured *in vivo*, related to NeanderScore and overlaid on the average skull of all 146 participants. Warm colors represent areas for which NeanderScore correlated with skull expansion (p < 0.05, FWE-corrected). There were no findings in the opposite direction. Right panel depicts previously published shape differences between Neanderthals and modern humans, from archeological samples, overlaid on a modern human skull. Red regions represent skull expansion in Neanderthals relative to modern humans; green represents contraction, adapted by permission from Macmillian Publishers Ltd: Nature^2, ©^ 2001. Note the convergence of *in vivo* and archeological data in occipito-parieto-temporal regions (arrows).

In line with the contention that Neanderthal genetic load is biologically functional in modern-day humans, we first examined normalized anterior-posterior cranial length, one of the earliest reported parameters differentiating modern human and Neanderthal skulls^11^, and found this parameter to significantly correlate with NeanderScore (R = 0.240, p < 0.003). Examining morphometry of the entire skull in a data driven fashion, we also discovered that higher NeanderScore was positively and selectively associated with the regional size of a broad posterolateral area of the skull extending from the occipital and inferior parietal bones to bilateral temporal locales (T_max_ = 3.5, p < 0.0001, family-wise error (FWE)-corrected; Fig. 1, left and middle). The area of maximal change localized to the right lambdoid suture area, involving both the occipital and parietal bones, a finding that corresponds to published shape differences between modern humans and Neanderthals as documented in fossil samples (Fig. 1, right), thus establishing the validity of our approach. To ensure the results were not driven by the population structure of the cohort, we repeated this analysis while also controlling for the first 4 dimensions from a multidimensional scaling analysis and found that the pattern of association between NeanderScore and skull shape did not significantly change from the original analysis.

### Relationships between NeanderScore and Brain Morphology

**Figure 2:**
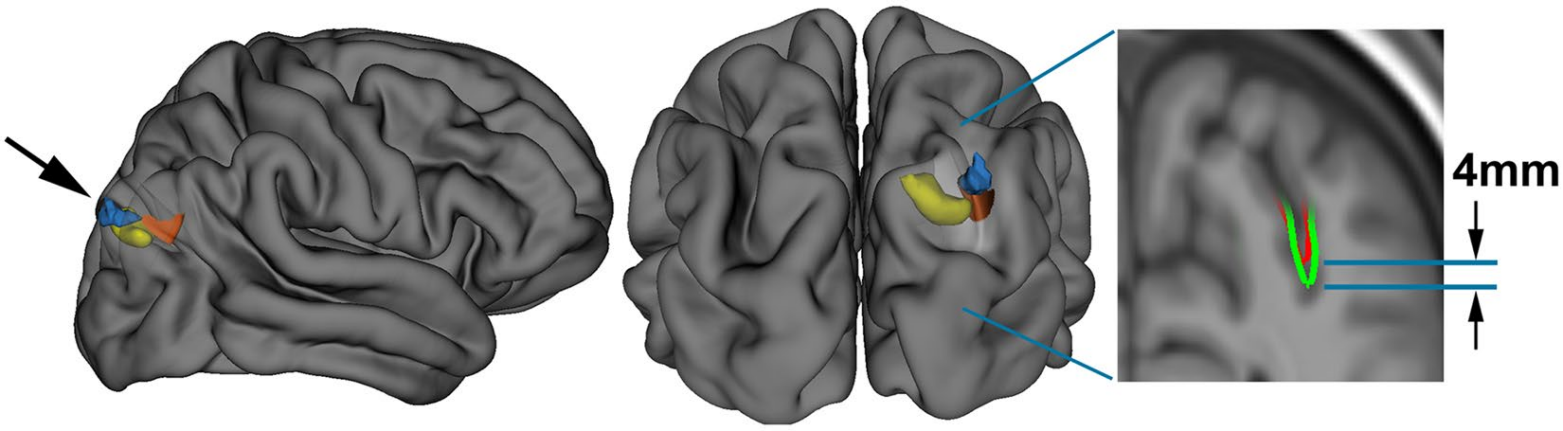
NeanderScore related brain changes in the intraparietal sulcus. Structural variation of the intraparietal sulcus (IPS) related to percentage of Neanderthal-derived SNPs (NeanderScore). Left and middle show lateral and posterior views of the right IPS on the average brain surface, illustrating the anatomical convergence of the associations of NeanderScore with greater sulcal depth (orange; p < 0.05 FWE-corrected), gray matter volume (blue; p < 0.005), and white matter volume (yellow; p < 0.005). Right shows a coronal slice (y = −72 mm) through the IPS where there was a sulcal depth difference of 4 mm between the 30 individuals with highest (blue line) and lowest (red line) NeanderScore. Findings are depicted on the average brain of all 221 individuals included in the study and underlie the maximal skull findings shown in Fig. 1 (arrows).

As previous work has related changes in skull shape to regional brain measures in European populations^23^, and having established that high NeanderScore is associated with statistically observable Neanderthal-like trends in modern cranial shape, we next sought to define contributions of Neanderthal-derived genetic variation to brain structure, as measured by regional sulcal depth and gyrification index, which we quantified across the entire cortical ribbon via segmentation and subsequent surface-based processing^24, 25^ of the full intracranial MRI dataset. We hypothesize that we might also finds similar NeanderScore-related changes in the brain regions underlying the NeanderScore-related occipito-parietal skull associations. The first measure we examined, regional sulcal depth, measures the extent to which each fold of the brain descends into the underlying mantle. We found that NeanderScore positively correlated with an increase in sulcal depth in the right intraparietal sulcus (IPS; T_max_ = 4.03, p_cluster_ < 0.001, FWE-corrected), directly beneath the area of maximal change in the prior skull shape analysis (Fig. 2), suggesting a significant but regionally circumscribed impact of Neanderthal genetic load on modern human brain.

**Figure 3:**
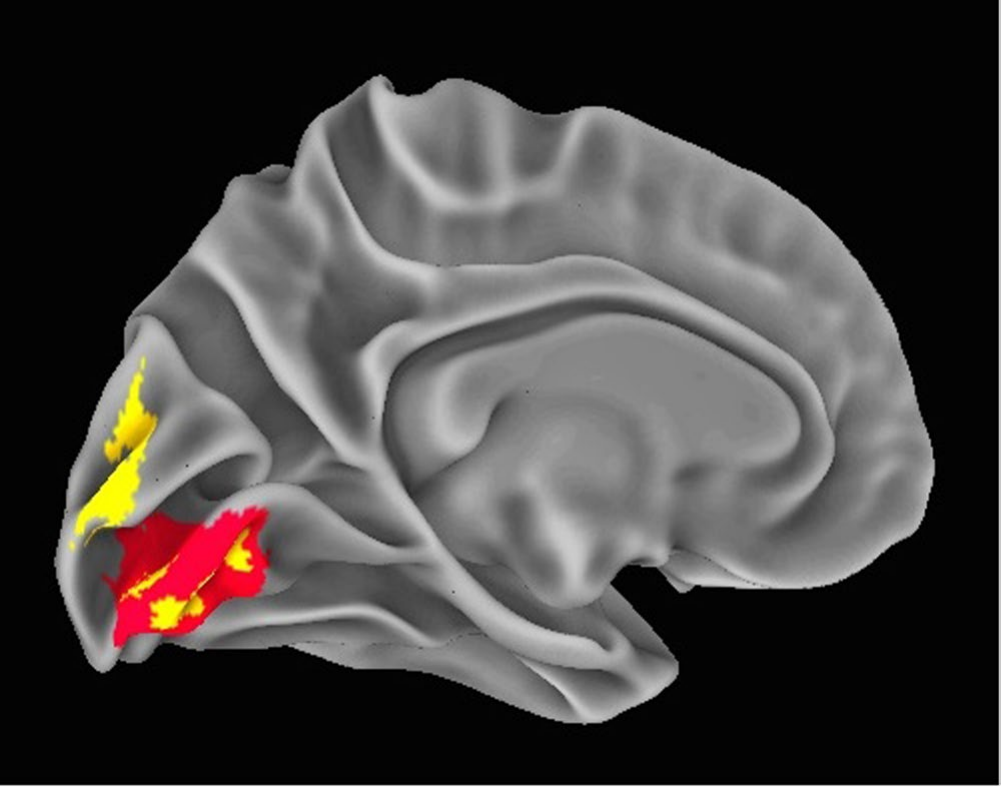
NeanderScore related brain changes in the primary visual cortex. Associations between local gyrification index, gray matter volume, and NeanderScore in visual cortex. Medial view of the primary visual cortex showing areas where NeanderScore was significantly associated with local gyrification index (red; p < 0.05 FWE-corrected) and with gray matter volume (yellow; p < 0.005).

The other examined neuroanatomic parameter, local gyrification index (LGI), provides a measure of the regional complexity of cortical folding, and is highly modified throughout evolution^26–28^. We found that NeanderScore positively correlated with LGI in early visual regions in the left hemisphere, also directly underlying the NeanderScore-associated skull findings, spanning primary visual cortex, V1 and V2 (T_max_ = 4.24, p < 3 × 10^−5^, FWE-corrected p < 0.0001; Fig. 3), as well as with the right superior temporal sulcus (T_max_ = 3.67, p < 3.1 × 10^−4^, FWE-corrected p < 0.005), such that cortical complexity, and thus cortical surface area, increased with NeanderScore.

The statistical strength and localization of NeanderScore associations with measures of cortical folding raised the possibility of additional structural differences in these brain areas. We therefore conducted follow-up, whole-brain, voxel-based morphometric analyses aimed at quantifying associations between NeanderScore and both gray and white matter volume across the brain. Associations with both gray matter volume (MNI_xyz_ = 28.5, −87, 36; T_max_ = 3.87, p < 1.4 × 10^−4^, uncorrected) and white matter volume (MNI_xyz_ = 18, −82, 28.5; T_max_ = 4.32, p < 2.4 × 10^−4^, uncorrected) were localized to a right parietal region adjacent to the IPS focus described above, such that greater Neanderthal SNP load correlated with more gray and white matter volume (Fig. 2). Similarly, in an area overlapping with the primary visual cortex gyrification finding described above, we observed that gray matter volume, though not white matter volume, was positively associated with NeanderScore (MNI_xyz_ = 0, −99, 10.5; T_max_ = 5.07, p < 8.5 × 10^−7^, uncorrected; Fig. 3). We also found evidence for an inverse correlation between NeanderScore and white matter volume in the orbitofrontal cortex (MNI_xyz_ = 37.5, 39; −7.5, T_max_ = −4.44, p < 1.4 × 10^−5^, uncorrected), a structure that appears to have been laterally restricted in Neanderthals relative to modern *H. sapiens* ^13^.

### Post-hoc Genetic Analysis of Neanderthal-Derived SNPs

**Figure 4:**
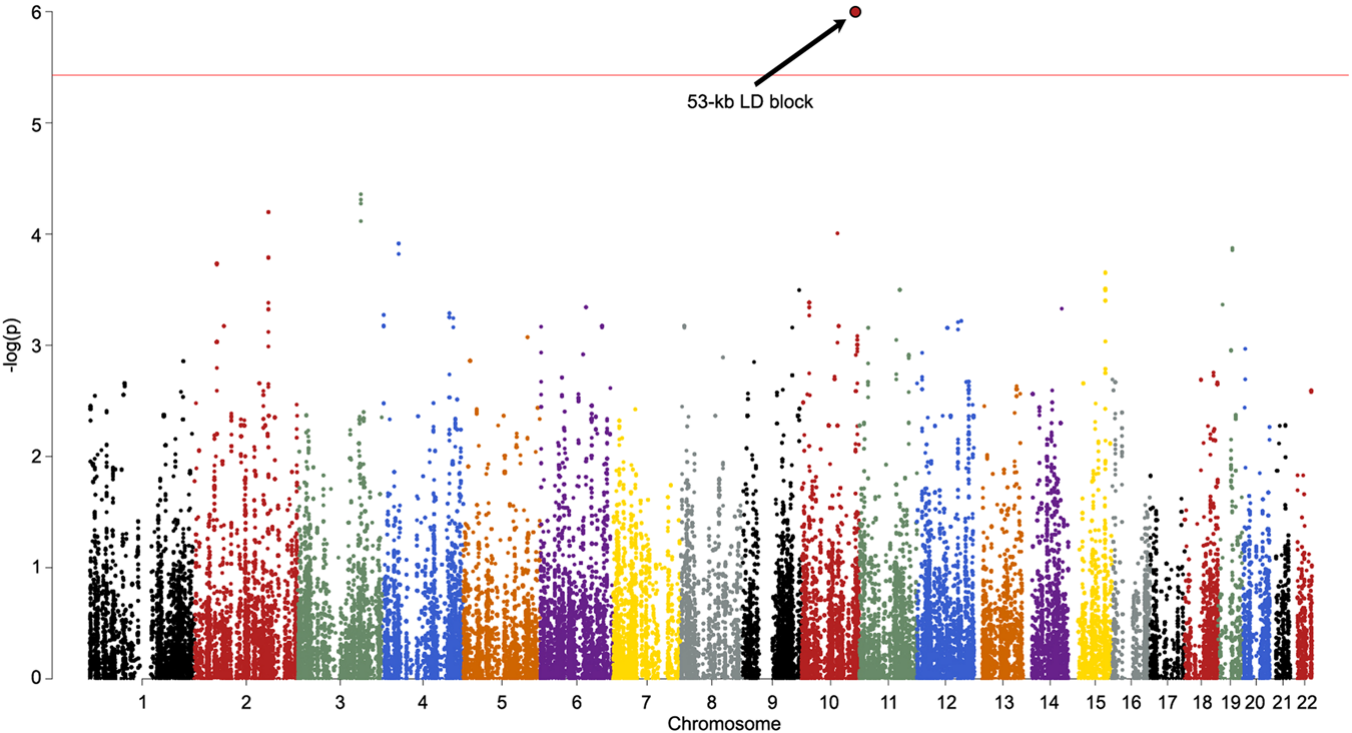
Manhattan plot of the shared variance of NeanderScore associated brain and skull changes. Manhattan plot showing the chromosomal position and significance of association between the shared variance of all Neanderthal-associated brain measures for each Neanderthal-derived SNP. Red line indicates Bonferroni-corrected threshold of p < 0.05 for the effective number of SNPs in the analysis. The arrow points to the significant 53-kb LD block on chromosome 10 containing 18 SNPs. See also Table 2.

**Figure 5:**
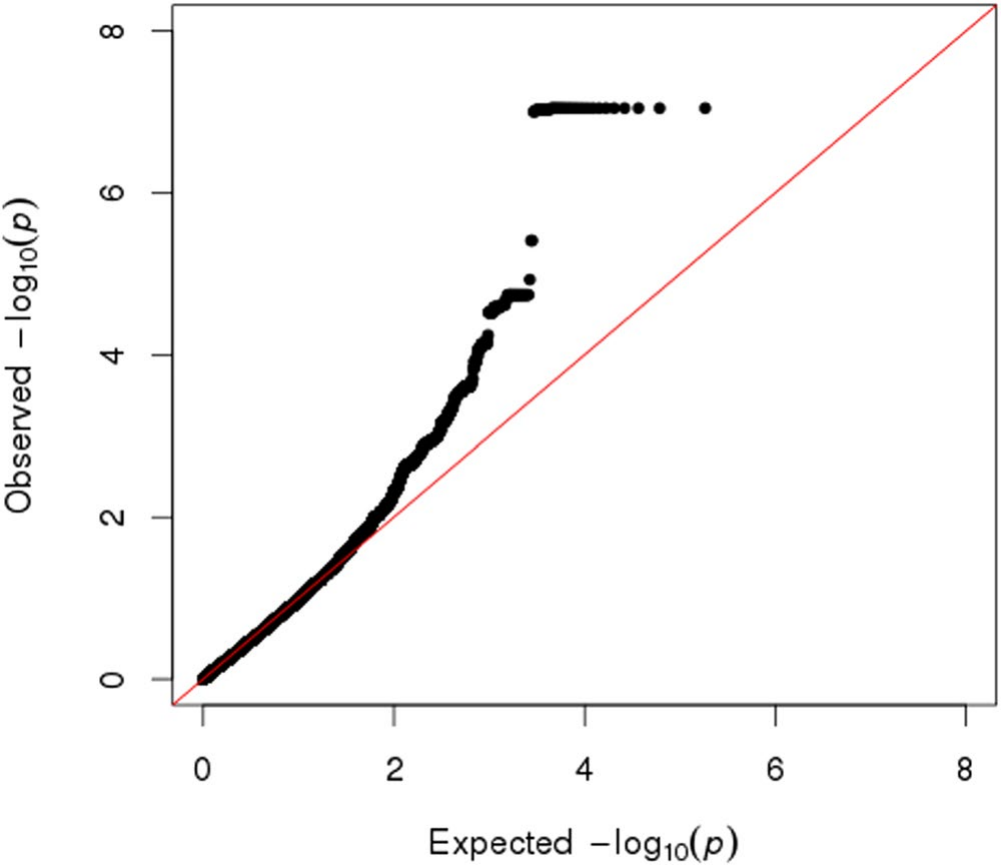
QQ plot showing observed versus expected −log_10_ (p values) for association at all Neanderthal-derived loci of the shared variance of all Neanderthal-associated brain measures.

Finally, we conducted an exploratory test of the association of our structural findings to specific variants within the 100,156 SNPs identified as putatively derived from Neanderthals in our participants. The convergence of findings in posterior head regions, across cranial, sulcal depth, gyrification and volumetric variables, led us to hypothesize that common genetic influences would be found in genes preferentially expressed in the human brain. Using the first component from a principal components analysis as a quantitative trait that captures the shared-variance across our structural measures, we tested each of the 100,156 Neanderthal-derived SNPs for association with brain/skull structure using a linear model. SNPs in a 53 kb LD-block of chromosome 10, encompassing the telomeric end of the gene *GPR26*, a G-protein coupled receptor preferentially expressed in the human brain^29^, showed strong association with brain/skull structure (t = 5.1, p < 1.04 × 10^−6^, Bonferroni-corrected p < 0.013, Table 2). Figure 4 shows the manhattan plot of these results and Fig. 5 shows the QQ plot.

**Table 2:** SNPs showing significant association with the composite brain/skull score.

Chromosome	hg19 BP Range	# of SNPs	SNPs	Uncorrected p-value	Bonferroni-corrected p-value	Associated Gene
10	125447351–125500316	18	rs77509056, rs34374660, rs17608529, rs17676917, rs17676960, rs17608655, rs17608690, rs117380193, rs59846519, rs4980201, rs75795353, rs150150087, rs143384481, rs4980208, rs80113622, rs79867143, rs4980157, rs4980255	1.037E-06	0.014	*GPR26*

We further examined this genetic region to assess the plausibility of its Neanderthal introgression. The 53 kb block we identified overlaps a previously predicted Neanderthal-introgressed region – by both Vernot *et al*.^30^ and Sankararaman *et al*.^5^. Since regions of extended similarity to archaic genomes could arise by incomplete lineage sorting (ILS), we calculated the maximum possible length of such an ILS region given the recombination rate and time since introgression^31^. Assuming the lowest average recombination rate in the region (which is most conservative), the branch lengths in years^8^, a generation time of 25 years, and a mutation rate of either 0.5 × 10^−9^ per site per year or 1 × 10^−9^ per site per year, we can rule out that any region longer than 1580 bp (0.5 × 10^−9^) or 3163 bp (1 × 10^−9^) is the result of ILS. The chance of maintaining a 53 kb block in both lineages is conservatively estimated at p < 9.4 × 10^−7^, using a gamma distribution with shape parameter 2 and a conservative rate parameter of 1/3163 bp^31^.

## Discussion

In this work, we describe relationships between Neanderthal-derived genetic variation and co-localized cranial and brain morphology in modern humans. The results show that greater NeanderScore is associated with more Neanderthal-like skull shape (corresponding to published shape differences between modern humans and Neanderthals as documented in fossil samples^2^ and shown in Fig. 1), as well as regional changes in brain morphology underlying these skull changes, specifically in the IPS and visual cortex. This work not only offers an unprecedented window into structure of the Neanderthal brain, but also characterizes the contributions of admixture with *H. neanderthalensis* to the evolution of the modern human brain.

In examining the associations of NeanderScore with skull shape, it is important to note that the topology of the identified occipito-parieto-temporal patch associated with NeanderScore specifically recapitulates the pattern of expansion in Neanderthal relative to anatomically modern human skulls previously reported from fossil remains (Fig. 1, right)^2^. This finding provides crucial validation of the NeanderScore measurement and suggests that even in the context of the modern *H. sapiens* genome, Neanderthal genetic variation is associated with patterns of skull dimensions that mirror known Neanderthal phenotypes.

Additional validation of the NeanderScore metric lies in its comparison with other reports characterizing Neanderthal-derived genetic contributions to modern humans. The coefficient of variation (i.e., the relative standard deviation, independent of the mean and comparable across measures) of the NeanderScore metric is consistent with previous published studies^5^ that were performed with a different goal: prior studies, unlike ours, sought to test for differences in admixture across populations, and have, therefore, calculated the proportion of the entire genome that was derived from Neanderthals. In those studies, approximately 1.15% of the entire genome of persons of European decent was found to be derived from Neanderthals (standard deviation = 0.08)^5^. Inherently, that approach and the present one capture related though different characteristics of Neanderthal-derived genetic information and the means (and therefore the standard deviations as well) of these differently derived measures are not the same. Importantly, however, the coefficient of variation in previous studies was also 0.070,^5^ identical to the coefficient of variation reported here, providing further validity to the NeanderScore measure.

In determining NeanderScore related changes in brain morphology, we found two significant cortical regions, the IPS and primary visual cortex, which both directly underlie the region of skull shape associations. The IPS, though present throughout highly gyrified modern primates^28^, has been theorized to have undergone substantial evolutionary expansion in hominids, with cross-species functional neuroimaging demonstrating unique visuospatial processing characteristics in modern humans relative to rhesus monkeys^32^. Additionally, cranial vault analyses of fossil skulls have suggested differences in the parietal lobes of Neanderthals^14, 15^, and the intraparietal sulcal region in particular has been hypothesized to be a focus for some of these differences^33^. Moreover, the fact that the IPS is particularly critical for tool manipulation in modern humans^34^ makes this finding even more intriguing in view of continued debate over the nature and development of Neanderthal tool use^35^.

The other brain region revealed to have significant associations with NeanderScore was primary visual cortex. This cortical region is responsible for the first steps in processing of visual information in the mammalian cortex and feeds into later brain regions in the ventral and dorsal visual processing streams (which differentially subserve object recognition and visuospatial object location, respectively)^36^, with the IPS playing a prominent role in the latter. Though the functioning of the primary visual system is relatively conserved in primates, the size of primary visual cortex in modern humans is smaller than would be expected from brain volume^37^. Our data not only suggest that this may be less the case in Neanderthals, but also are consistent with cranial remains showing more prominent visual systems in Neanderthals than in modern humans^15^.

It should be noted that we did not find associations of NeanderScore with smaller frontotemporal volumes^38^ or shortened anterior extension of the temporal lobes^13^, as might have been hypothesized from previous cranial analyses of *H. neanderthalensis*, suggesting either that these particular phenotypes, if accurate, are not driven by the allelic variation captured by the NeanderScore measurement, or that such effects are more directly influenced by any of the myriad factors that establish the genetic and biological context of this modern cohort. Additionally, some of these phenotypes may be only partially modulated by genotypic factors inherited from Neanderthals, with an effect too small to be observed in our sample. In contrast, the effect sizes we observed here for the associations of NeanderScore with the skull and brain measures were moderate (average Cohen’s d = 0.58) but appropriate for the sample sizes used.

The analyses reported here were restricted to a sample of individuals of European descent. It is known that the degree of admixture is variable in different modern populations. For example, East Asian populations have been found to have a larger portion of the genome derived from Neanderthals than European populations (up to 20% more), though the admixed regions of the genome are not necessarily overlapping^5^. This raises the possibility that the findings reported here may not translate to other populations, where Neanderthal introgression may involve other genomic regions that may be functional in different ways. As large neuroimaging and genetic data from different populations become available, future work could investigate this possibility by performing similar analyses in different populations, including using African populations with minimal Neanderthal admixture as potential “null hypothesis” groups.

Finally, our analyses of the relationship of our findings to specific Neanderthal-derived gene variants, revealed a single 53 kb LD block that was significantly associated with the shared variance of the identified Neanderthal-derived brain and skull changes and that encodes for the gene *GPR26*. In line with our primary hypothesis for this analysis, that such genetic influences would be found in genes preferentially expressed in the human brain, *GPR26* in fact encodes a G-protein coupled receptor subtype that is preferentially expressed in the brain^29^. Interestingly, in human post-mortem brains samples, expression of *GPR26* peaks perinatally^39^, when the visual system is first challenged, indicating that it may play a role in development of the human visual system. Mouse models also show this gene to impact both affective and energy homeostatic functions^40, 41^. Additionally, this G-protein-coupled receptor has been shown to form oligomers with the 5-HT1a receptor^42^, perhaps providing a putative mechanism underlying the Neanderthal-related brain changes found here. Although the nature of the influence of this region on modern and archaic human nervous systems is uncertain, a possible link between brain energy regulation, neurodevelopment and mature structure may merit further investigation.

Because the NeanderScore measure employed here is, itself, polygenic, it is likely that the genetic contributions to skull and brain morphology we observed involve a number of different genetic loci. Nonetheless, our exploratory post-hoc genome-wide analysis of the shared variance of these findings identified only a single significant region. It is unlikely that this single locus, the LD block on chromosome 10, fully explains the brain and skull findings, and in fact, the Manhattan plot in Fig. 4 suggests that multiple other regions that do not meet strict Bonferroni corrections may represent some degree of true signal. Our modest sample size may have been underpowered to identify these additional signals. As larger datasets containing both genotype and neuroimaging data of brain and skull become available, future work will likely uncover additional genetic regions contributing to these findings.

Taken together, the associations between Neanderthal sequence variation and co-localized skull and brain morphology in modern humans engender an enduring, living footprint of *H. neanderthalensis* – a residual echo of shared, intimate history with a fallen lineage close to our own. To the extent that characterization of Neanderthal variation in present-day people can provide insights into archaic human phenotypes, this work can form the basis of future studies aimed at a more thorough understanding of Neanderthal biology. By the same token, we suggest that Neanderthal gene flow into modern humans is not only of evolutionary interest, but may also be functional in the living *H. sapiens* brain, revealing novel genetic influences on neurodevelopment of the visuospatial system upon which a fuller account of molecular mechanisms of IPS-driven normative mental functions, such as visuospatial integration and tool manipulation, can be built. This, in turn, may inform models of IPS-associated cognitive disability as seen in select developmental and neurological disorders^43–46^.

## Methods

### Subjects

Two hundred twenty-three participants were identified from a pool of healthy control volunteers recruited as part of the NIMH Sibling Study – a multidisciplinary initiative aimed at examining the neurobiology of schizophrenia risk – who had completed both neuroimaging with 3-Tesla MRI and genome-wide SNP genotyping. Participants included in the current work were all adults of self-reported European-descent and were screened to be healthy and free of any neurologic or psychiatric diseases based on clinician-obtained history and physical examination, semi-structured diagnostic interview (Structured Clinical Interview for DSM-IV), clinical MRI of the brain, and routine laboratory testing, including urine toxicology. Table 1 provides the subject demographics. All procedures were carried out in accordance with NIH guidelines and were approved by the National Institutes of Health CNS Institutional Review Board, and all participants provided written informed consent for the study procedures.

### Genetic Analysis

Genotyping was carried out on DNA extracted from lymphoblast cell lines derived from each individual. As data from this study has been collected over time, genotyping was performed in four steps with an increasing number of SNPs genotyped at each step. All genotyping was done on Illumina QUAD SNP chips (ranging from 550K-2.5 M SNPs). The 275,659 common SNPs from all chip types were combined and Illumina genotype QC and imputation procedures were performed following the previously reported methods^47^. After QC procedures, data was pre-phased with SHAPEIT, imputation was done with IMPUTE2, using 1000 genome phase 1 data as a reference panel. Ambiguous SNPs and duplicated SNPs were removed prior to imputation.

To ensure participants were of similar genetic ancestry, clustering and multidimensional scaling was performed in Plink (http://pngu.mgh.harvard.edu/~purcell/plink), using a set of 125 ancestry informative markers^17^. Two individuals were noted to be of genetically different ancestry, based on the pairwise identity-by-state distance information of all participants and appeared to cluster separately from the rest of the group on plots of the first two principle components of the multidimensional scaling, and were removed from further analysis.

### Calculation of NeanderScore

Previous studies aimed at examining Neanderthal ancestry have identified the proportion of the genome derived from Neanderthals, predominantly to examine differences in admixture across populations^5^. Here, instead of examining for population effects, we sought to quantify the proportion of potential Neanderthal admixture in each individual by calculating an enhanced D statistic. Enhanced D statistics have been shown to be linearly related to the amount of Neanderthal ancestry in an individual^48^ and are termed “enhanced” because they improve the signal to noise ratio of basic D statistics by restricting computation to sites where an isolated population carries the ancestral allele^9^. This approach could be interpreted as a polygenic score for Neanderthal inheritance, similar to other scores used to study the genetics of disease^49^. To accomplish this, we sought to identify single nucleotide polymorphisms (SNPs) derived from Neanderthals via admixture. Admixture may have occurred at a SNP location if (1) the derived (non-ancestral) allele is present in both humans and Neanderthals, (2) the genotyped individual carries the derived allele, and (3) that derived allele is absent in a human population devoid of Neanderthal admixture^5^. In our case, we specifically identified SNPs in the genome at which Neanderthal genotyping indicates the presence of a derived allele that is not present in a population without admixture, based on publically available data that has previously been used to estimate Neanderthal ancestry^5^. As in that previous work, we used the high coverage Altai Neanderthal genome^8^ as the Neanderthal sequence, the 1000 genomes project Yoruba population^50^ as the ancestral human population devoid of admixture with Neanderthals, and a 6-primate consensus sequence^51^ to represent the ancestral species.

**Figure 6:**
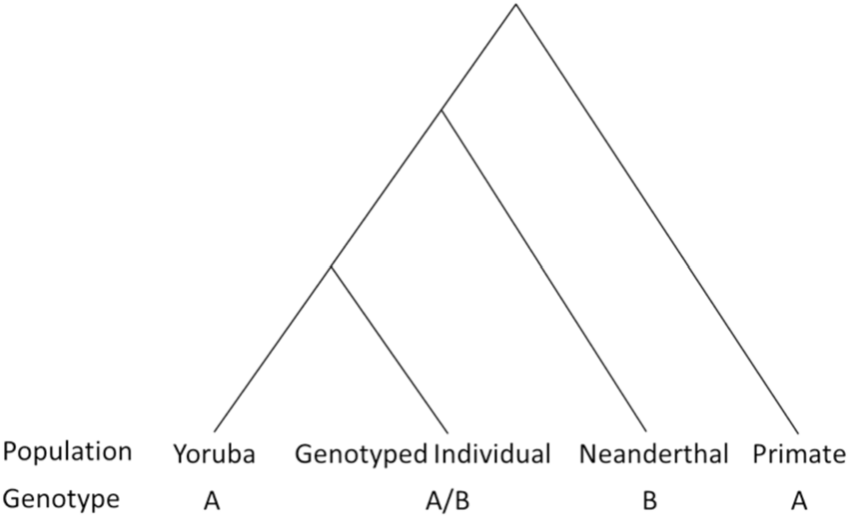
Derivation of NeanderScore. Phylogenic tree showing relationship of Yoruba, a genotyped individual, Neanderthal and Primates. At a given SNP, Yoruba and Primates contain the ancestral allele (**A**), Neanderthal contains the derived allele (**B**) and the genotyped individual may have either allele (**A**,**B**).

We determined a “NeanderScore” for each genotyped individual, which is defined as:

$$Neander\,Score=\frac{{\sum }^{}({n}_{ABBA})}{{\sum }^{}({n}_{ABBA})+{\sum }^{}({n}_{AABA})}$$

where ∑(*n*_*ABBA*_) is the count of SNP locations at which the genotyped individual shares an allele with the Neanderthal sequence but differs from all Yoruba and primate sequences, and ∑(*n*_*AABA*_) is the count of locations at which the genotyped individual shares an allele with Yoruba and primates sequences but differs from the Neanderthal sequence. Importantly, the denominator in this equation represents all SNPs where the Neanderthal sequence differs from (1) all individuals in the Yoruba population and (2) the consensus primate sequence (i.e., all SNPs that could be tested for contribution from Neanderthals). The numerator is the number of those sites in which Neanderthal contribution was found in a particular individual. The ratio, or NeanderScore, represents the percentage of potential Neanderthal-derived SNPs in that individual, whereas prior reports have estimated the percentage of the entire non-African genome derived from Neanderthals^3, 5, 8^. Figure 6 depicts this graphically in a phylogenic tree where genotype ‘A’ represents the ancestral allele, present in both Yoruba and the primate consensus, and genotype ‘B’ represents the derived allele, assumed to have passed from the Neanderthals to the genotyped individual.

### Image Acquisition and Preprocessing

Three-dimensional structural MRI scans were acquired on a 3-Tesla GE scanner (GE Medical Systems, Milwaukee, WI) using a magnetization prepared rapid gradient echo (MPRAGE) sequence (repetition time 7.28 ms, echo time 2.74 ms, 120–136 slices, resolution 0.859 × 0.859 × 1.2 mm). Preprocessing included intensity nonuniformity normalization^52^ and rigid alignment to MNI space.

### Skull Surface Reconstruction

**Figure 7:**
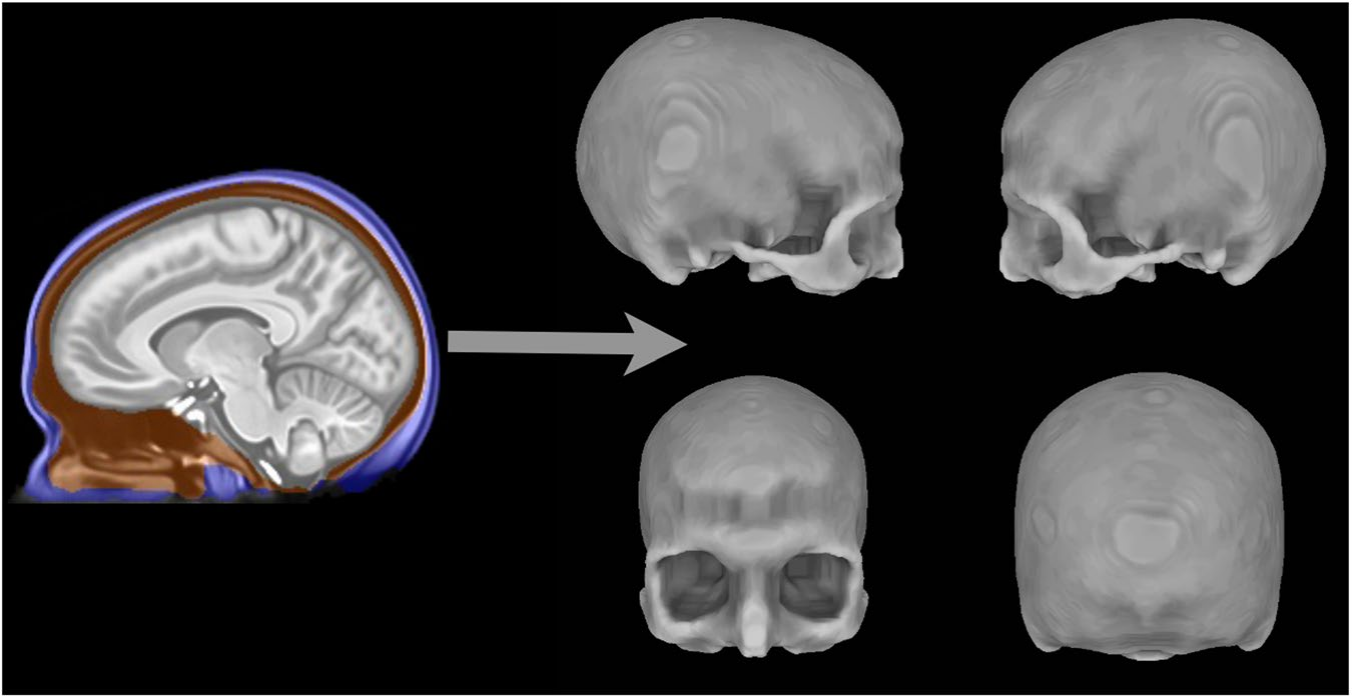
Skull surface creation from the T1-weighted MRI scan of a single participant. Left shows the segmentation procedure performed in the volume where **v**oxels labeled blue represent scalp, orange voxels represent skull, and gray voxels represent the intracranial area. Right shows the 3D skull surface derived from voxels labeled as skull (orange) in the segmentation.

All images were visually inspected to ensure that the entire head was represented. Of the 221 participants’ scans, 146 were deemed usable for skull reconstruction. We applied previously published methods^19–21^ to our participants’ MRI data after using SPM8 (http://fil.ion.ucl.ac.uk/spm) to perform segmentation of individual scans into tissue probability maps, representing voxel-wise probabilities of gray matter, white matter, cerebrospinal fluid (CSF), soft tissue, bone or “other.” After identifying head and intracranial masks for each participant, skull voxels were identified as those that (1) were within the head mask, (2) were outside of the intracranial mask, and (3) had a combination of high bone-tissue probability and low probability of gray matter, white matter and CSF. An example of these boundaries is shown in Fig. 7 (Left). Individual volumes representing skull voxels were used to produce 3D surface representations (meshes) of participants’ skulls, using SUMA tools (http://afni.nimh.nih.gov/afni/suma/). An example skull surface is also shown in Fig. 7 (Right).

### Skull Surface Analysis

A group-average template of all 146 MRI scans used in the skull analysis was created using ANTs software (http://stnava.github.io/ANTs). The skull surface of this group average template was created as described above for the individual scans. Next, each individual scan was aligned to the template using nine-degrees of freedom (accounting for shifts, rotations and scaling about the x-, y- and z- axes) to normalize for skull size without altering shape. Using SUMA tools, the signed Euclidian distance between each node of the template and the nearest node on the surface of each individual skull was calculated. Areas for which skull shape co-varied with NeanderScore, controlling for age and sex, were determined based on previously reported methods^22^. As neuroimaging data are highly dimensional and statistical analyses may be biased towards type-I error, a surface-based family-wise error correction for multiple comparisons was calculated using permutation testing on 10,000 Monte-Carlo simulations of synthesized Gaussian white noise with the structure and smoothness of the underlying data^53, 54^. Resulting statistical maps were thresholded at p < 0.05, corrected for multiple comparisons using this family-wise error (FWE) correction. Additionally, the anterior to posterior distance for each individual skull was calculated using SUMA tools and was correlated with NeanderScore using IBM SPSS Statistics for Windows, version 21.0 (SPSS, IBM Corp, Armonk, NY) after controlling for effects due to age and sex.

### Cortical Surface-based brain analyses

The 221 individual brain scans were processed with the FreeSurfer version 5.3 pipeline (http://surfer.nmr.mgh.harvard.edu) to produce surface-based maps of sulcal depth and local gyrification index for each individual. The surface-based maps were then aligned to a 198,812 node standard mesh^55^. Statistics were computed for each node on a participant’s surface, correlating sulcal depth and local gyrification measures with NeanderScore while controlling for age and sex. A surface-based correction for multiple comparisons was determined using the methods described above for skull shape analysis, with resulting maps thresholded at p < 0.05, corrected for multiple comparisons using FWE correction^53, 54^.

### Voxel-based brain analyses

After the image preprocessing described above, voxel-wise tissue density maps, including gray and white matter maps, were computed using SPM8’s segmentation functions. SPM8’s DARTEL tools were used to create a group-average template. Voxel-based morphometry was performed, based on tissue density maps modulated by the jacobian determinant of participants’ deformation maps to create voxel-wise volumetric maps of each tissue class^56^. Maps of gray matter volume and white matter volume were spatially smoothed using a 6 mm FWHM Gaussian kernel. Linear regression was performed on the resulting maps on a voxel-wise basis, using NeanderScore as a regressor of interest, while controlling for age and sex effects. Resulting statistical maps were thresholded at a significance level of p < 0.005, uncorrected.

### Post-hoc genetic analyses

Post-hoc analyses were conducted to determine whether individual Neanderthal-derived SNPs were of particular importance in influencing converging skull/brain variation found within the intraparietal area and the primary visual cortex in modern humans. We pre-processed the peak results from the skull, IPS sulcal depth, primary visual local gyrification index, IPS/primary visual gray matter volume and IPS white matter volume analyses by removing effects due to age and sex from each result individually using linear regression. We then conducted a principal components analysis using SPSS on the residualized variables, using the first principal component to form a composite score representing the primary inter-individual variance shared across the skull/brain measures. This composite served as a quantitative phenotype in a linear genetic model using Plink (http://pngu.mgh.harvard.edu/~purcell/plink) to examine each Neanderthal-derived SNP. GEC (http://statgenpro.psychiatry.hku.hk/gec) was used to determine the number of effective SNPs in this analysis (13378 SNPs)^57^, yielding a Bonferroni correction level of p_corrected_ < 0.05 occurring when p_uncorrected_ < 3.7 × 10^−6^.
